# Comparative Mitochondrial Genomics within and among Yeast Species of the *Lachancea* Genus

**DOI:** 10.1371/journal.pone.0047834

**Published:** 2012-10-24

**Authors:** Anne Friedrich, Paul P. Jung, Jing Hou, Cécile Neuvéglise, Joseph Schacherer

**Affiliations:** 1 Department of Genetics, Genomics and Microbiology, University of Strasbourg, CNRS, UMR7156, Strasbourg, France; 2 INRA, UMR1319 Micalis, Thiverval-Grignon, France; Institut de Genetique et Microbiologie, France

## Abstract

Yeasts are leading model organisms for mitochondrial genome studies. The explosion of complete sequence of yeast mitochondrial (mt) genomes revealed a wide diversity of organization and structure between species. Recently, genome-wide polymorphism survey on the mt genome of isolates of a single species, *Lachancea kluyveri*, was also performed. To compare the mitochondrial genome evolution at two hierarchical levels: within and among closely related species, we focused on five species of the *Lachancea* genus, which are close relatives of *L. kluyveri*. Hence, we sequenced the complete mt genome of *L. dasiensis*, *L. nothofagi*, *L. mirantina*, *L. fantastica* and *L. meyersii*. The phylogeny of the *Lachancea* genus was explored using these data. Analysis of intra- and interspecific variability across the whole *Lachancea* genus led to the same conclusions regarding the mitochondrial genome evolution. These genomes exhibit a similar architecture and are completely syntenic. Nevertheless, genome sizes vary considerably because of the variations of the intergenic regions and the intron content, contributing to mitochondrial genome plasticity. The high variability of the intergenic regions stands in contrast to the high level of similarity of protein sequences. Quantification of the selective constraints clearly revealed that most of the mitochondrial genes are under purifying selection in the whole genus.

## Introduction

Yeasts and more precisely the phylum of Hemiascomycetes have significantly contributed to have a better understanding of mitochondrial (mt) genome evolution of species spanning a broad distance. The complete mitochondrial genome sequence is now available for more than 40 hemiascomycetous species, which are representative of different genera [1]. This ever-growing data represented an opportunity to have a deep insight into the mtDNA organization and the genome architecture variation from an evolutionary perspective in a whole phylum.

To obtain a global view of the genetic variations occurring in the mtDNA within a species, we recently performed a genome-wide polymorphism survey on the mt genome of 18 *Lachancea kluyveri* (formerly known as *Saccharomyces kluyveri*) isolates [2]. We generated a comprehensive view of mitochondrial sequence polymorphism in this single species. Interestingly, the comparison revealed that the genomes are syntenic, but the size of the mtDNA differs. In addition, the whole genome analysis clearly showed a higher rate of SNPs and indels in the intergenic regions compared to the coding regions. Selection was evaluated and the results clearly suggested that purifying selection purged most indels and non-synonymous differences from mitochondrial protein-coding genes.

In this study, we decided to explore the organization, selection and architecture variation of mitochondrial genomes in close relatives of the *L. kluyveri* species. Our purpose was to examine and compare the mitochondrial DNA sequence variation and evolution at two hierarchical levels: within and among closely related species. Such analyses are of interest and importance because it allows having an insight into the mitochondrial genome variation over evolutionary time, never explored so far. We therefore focused on the mitochondrial genome of a single isolate from five different species of the *Lachancea* genus.

In 2003, the genus *Lachancea* was proposed by Kurtzman to accommodate a small group of species from different genera (*Zygosaccharomyces*, *Kluyveromyces* and *Saccharomyces*), which rRNA sequences are close to each other to different extent [3]. Species of the *Lachancea* genus are found all over the world and inhabit many niches including soil, plants, insects but also processed food and beverages [4]. In addition, *Lachancea* species are protoploid *Saccharomycetaceae*, which means that they diverged from the *S. cerevisiae* lineage prior to undergoing ancestral whole genome duplication (WGD) and are therefore a pre-WGD yeast species [5].

Here, we report the complete mt genome sequence of five *Lachancea* species: *L. dasiensis* (CBS 10888), *L. nothofagi* (CBS 11611), *L. mirantina* (CBS 11717), *L. fantastica* (CBS 6924) and *L. meyersii* (CBS 8951) [6], [7], [8], [9]. These strains were isolated from various geographical locations and ecological niches (seawater, soil, plants, and distillery). We first sequenced, assembled and annotated the mt genomes. To provide a better picture of the mtDNA evolution in the *Lachancea* genus, we compared these mt genomes with the previously sequenced mt genomes of *L. thermotolerans* (CBS 6340) and *L. kluyveri* (CBS 3082) [10], [2]. We explored the phylogenetic relationship of the seven species. Whole-genome analysis clearly showed that there is a high degree of conservation of the gene content and the synteny at the two hierarchical levels: within and among closely related species. Nevertheless the mitochondrial genomes are variable in size, which is related to the variation of the intron content and the size of the intergenic regions. As previously seen in the *L. kluyveri* species, the dN/dS ratios clearly suggested that the protein-coding genes, with the exception of the *VAR1* gene, are under purifying selection in the whole genus.

## Materials and Methods

### Strains and DNA Preparation

Yeast species were mostly obtained from the Centraalbureau voor Schimmelcultures: *Lachancea meyersii* (CBS 8951, isolated from seawater of mangrove creek in the US), *L. fantastica nomen nudum* (CBS 6924, isolated from garden soil in South Africa), *L. nothofagi* (CBS 11611, isolated from *Nothofagus* in Patagonia), *L. dasiensis* (CBS 10888, isolated from leaf of *Angiopteris lygodiifolia* in Taiwan). *L. mirantina* (CBS 11717 =  CLIB 1160, isolated from a distillery in Brazil) was kindly provided by Serge Casarégola from the CIRM-Levures (http://www.inra.fr/cirmlevures/).

**Figure 1:**
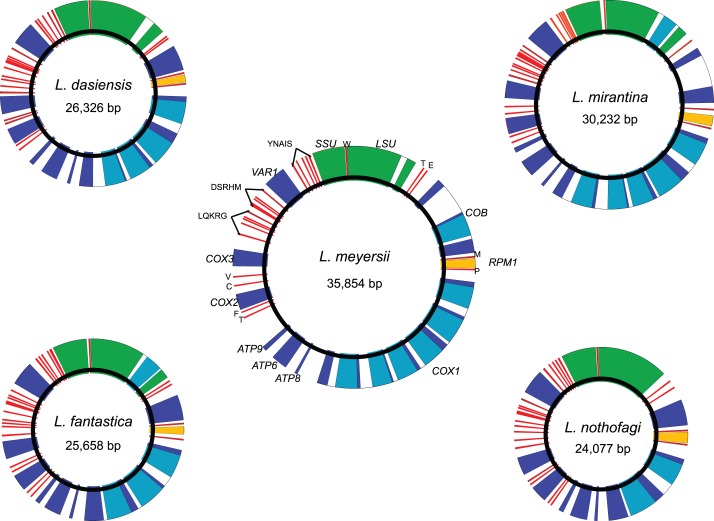
Circular maps of the mitochondrial genomes. Protein-coding genes, tRNA and rRNA genes are presented in dark blue, red and green, respectively. Coding introns are represented in light blue, and non-coding introns in white. The *RPM1* gene is presented in yellow.

**Table 1:** General features of the mitochondrial genomes of the *Lachancea* genus.

Species	Strains	Size (bp)	GC%	Coding region (%)	Intergenic region (%)	Intron number	Total intron size (bp)	References
*L. thermotolerans*	CBS 6340	23,584	24.8	28	24.2	3	4,320	Talla *et al.* 2005
*L. kluyveri*	CBS 3082	51,525	15.1	12.8	58	6	8,777	Jung *et al.* 2012
*L. meyersii*	CBS 8951	35,854	22.1	18.4	30.4	9	11,477	This study
*L. fantastica nomen nudum*	CBS 6924	25,658	24.7	25.7	27.6	4	5,354	This study
*L. nothofagi*	CBS 11611	24,077	23.7	27.4	31.1	2	2,943	This study
*L. dasiensis*	CBS 10888	26,326	21.3	25	26.2	5	5,966	This study
*L. mirantina*	CBS 11717	30,232	19.8	21.9	23.1	7	9,786	This study

Cultures were grown on YPD at 28°C. Cells were collected during exponential growth, lysed with zymolyase 100T (ICN Biochemicals, Aurora Ohio USA) and treated with SDS. For each species, mitochondrial DNA was separated from nuclear DNA by centrifugation in a CsCl-bisbenzimide gradient.

### Sequencing and Assembly

Genomic paired-end Illumina sequencing libraries were prepared and multiplexed in an Illumina HiSeq 2000 lane for sequencing. Paired-end reads from 104 nt, 6 of which were dedicated to the multiplex tag, were obtained. FASTX-Toolkit (http://hannonlab.cshl.edu/fastx_toolkit/) was used to clean the reads, with “−t 20 −l 50” options. Several independent *de novo* assemblies were then constructed using SOAPdenovo version 1.05 [11], with different Kmer sizes (-K 57, -K 63 and -K 75), using a subset of 250,000 reads for each isolate.

**Figure 2:**
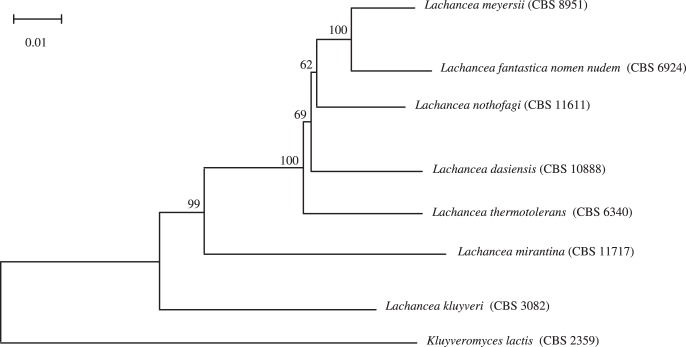
Neighbor-Joining tree based on the concatenation of mt genes, showing the relationship of species of the *Lachancea* genus. *K. lactis* is used as the outgroup.

For each species, mitochondrial contigs and scaffolds were identified by similarity searches with the BLAST suite of programs [12], using the *L. kluyveri* (CBS 3082 strain) mitochondrial genome as query [2]. The highlighted sequences were compared with MUMmer 3.0 [13] to detect overlapping segments and the alignment of all these sequences was refined manually in order to obtain a single contig per species.

**Figure 3:**
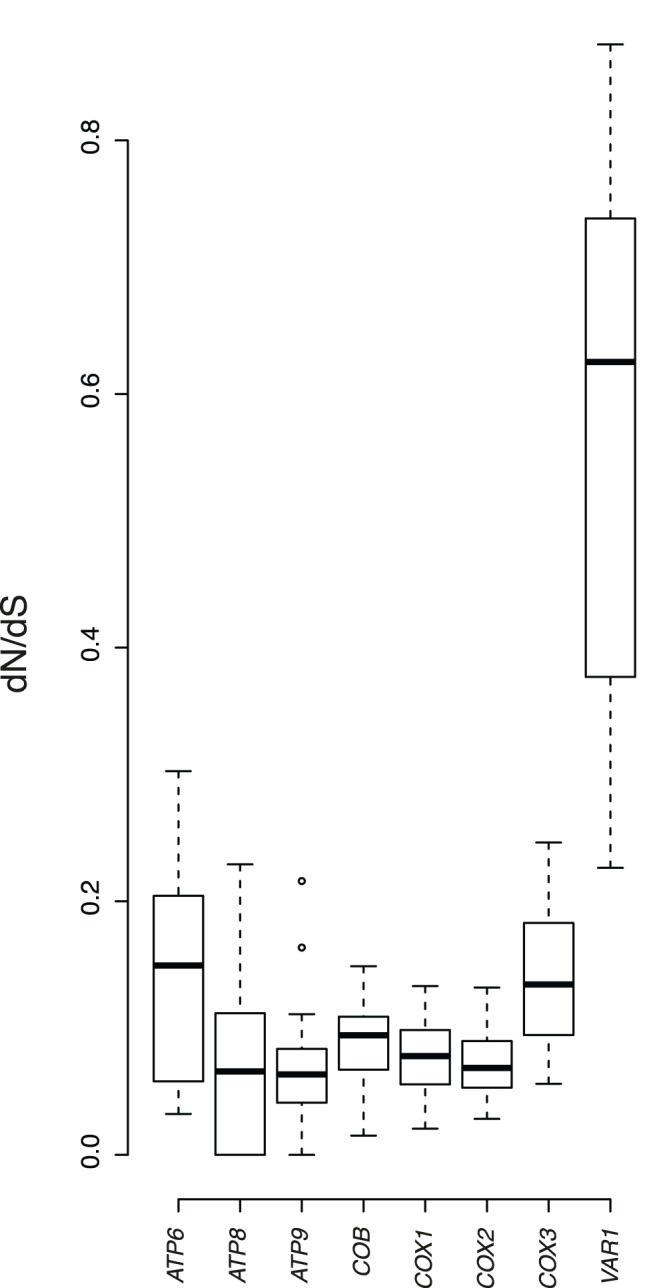
Box-plot comparisons of the dN/dS ratio [ω] estimated in the various mt genes and based on pairwise alignments.

Sequences are available under EMBL accession numbers HE983614 for the CBS 8951 strain, HE983610 for the CBS 6924 strain, HE983612 for the CBS 11611 strain, HE983611 for the CBS 10888 strain and HE983613 for the CBS 11717 strain.

### Gene Annotation

Similarity searches were performed, with *L. kluyveri* gene sequences as query, to localise the protein-coding genes as well as the large and small subunits of the ribosomal RNA genes on the complete mitochondrial sequences.

The *COX1* and *COB* intronic regions were refined manually based on the conservation of the protein sequences. The position of introns within the *LSU* genes was determined based on the boundaries defined for both *S. cerevisiae* and *L. thermotolerans* species [14]. tRNA genes were assigned combining two programs: TRNAscan-SE and RNAweasel [15], [16].

### Phylogenetic Studies

Multiple alignments of the coding sequences of each protein-coding gene (*ATP6*, *ATP8*, *ATP9*, *COB*, *COX1*, *COX2, COX3* and *VAR1*) were generated with MUSCLE [17]. Each alignment was manually inspected before concatenation.

Based on these 6711 aligned positions, phylogenetic relationships among the *Lachancea* species were analyzed using Neighbor-Joining method, with the Kimura 2-parameter substitution model. Bootstrap analyses (1000 replications) were used to assess the confidence level of each node for the Neighbor-Joining method.

### Selection and dN/dS Ratios

The dN/dS ratios were calculated using CODEML model in PAML package version 4.4b [18]. We used a tree-based maximum-likelihood method. Briefly, coding sequence multi-alignments was generated for each of the 8 protein-coding genes among the different species, as described before. Based on the alignments, Neighbor-Joining trees were constructed using ClustalX and then labelled manually considering the branch-length and genetic proximity of the strains. Estimate of the dN/dS ratios were then calculated for each gene.

## Results and Discussion

### Genome-wide Characteristics

*De novo* assembly of the reads yielded a single contig for each of the five mt genomes (*L. meryersii*, *L. fantastica*, *L. nothofagi*, *L. dasiensis* and *L. mirantina*). The general features of these mtDNA are given in Table 1. All genomes can be mapped as circles, considering the sequences present at both ends of each contig (Fig. 1). Nevertheless, they may potentially exist as linear concatemers *in vivo*. In *S. cerevisiae* nearly all of its mtDNA is organized in polydisperse linear tandem concatemers, accompanied by small amounts of circular forms [19]. The sizes of genomes vary from 24,077 bp in the case of *L. nothofagi* to 35,854 bp in that of *L. meryersii*. Despite this size variation, all the five mtDNA encode for the same core of 35 genes. All genes are transcribed from one DNA strand. Such as the mtDNA of *L. kluyveri* and *L. thermotolerans* previously sequenced, this set of genes is composed of 8 protein-coding genes and 27 non-coding RNA genes [10], [2]. Protein-coding genes encode 3 subunits of the cytochrome c oxidase (*COX1*, *COX2* and *COX3*), 3 subunits of the ATP synthase (*ATP6*, *ATP8* and *ATP9*), the apocytochrome b (*COB*) and a ribosomal protein (*VAR1*). The non-coding RNA genes comprise two genes encoding the small and large RNA subunits of the ribosome (*SSU* and *LSU*, respectively), the *RPM1* gene that codes for the RNA subunit of the RNaseP and 24 tRNA genes. This set of tRNA genes includes at least one tRNA for each of the 20 amino acids and sufficed to decipher the mitochondrial genetic code. By contrast to several species distantly related to *S. cerevisiae*, mt genomes of the *Lachancea* genus do not encode the seven subunits of the NADH: ubiquinone oxidoreductase complex (complex I) [20], [21]. These genes are absent from mtDNA of the species of the *Saccharomycetaceae* family [22].

### Synteny is Conserved Across the *Lachancea* Genus

Complete analysis of gene order conservation between *Lachancea* species showed that these sequences are completely syntenic (Fig. 1). Only *L. kluyveri* possesses a translocation of the two tRNA genes surrounding the *COX2* gene, which breaks the synteny. This conservation is unusual and is not a general rule across the mtDNAs sequenced so far. The synteny varies considerably within Hemiascomycetes, which reflects small rearrangements occurring in the history of hemiascomycete fungi. As an example, gene order is highly rearranged in the mt genomes of yeasts of the *Nakaseomyces* clade [23]. Inversion as well as movement of short intergenic repeat and mobile introns seems to be at the origin of this variability [24]. Conservation of synteny seems to be correlated with the topology of the mtDNA. In fact, rearrangements might be more frequent in circular than in linear mitochondrial genomes [25]. Nevertheless, there is a couple of exception regarding the conservation of synteny. It has been shown a high conservation of synteny between closely related species such as *C. parapsilosis*, *C. orthopsilosis* and *C. metapsilosis* [26]. More recently, the genomes of the species of the *Yarrowia* clade were also found to be completely syntenic. This observation might suggest that these species are closely related [20].

In the case of the mt genomes of the *Lachancea* genus, the conservation of synteny might also reflect a close relationship between these species. Karyotypic analysis of different *Lachancea* species was recently performed [4]. All these species seem to have the same number of chromosomes, eight, pointing out the evolutionary relationship between them. Nevertheless, this analysis also revealed a significant size polymorphism of chromosomes, showing that nuclear chromosomal rearrangements occurred in this genus. Interestingly, this observation clearly indicates that mitochondrial and nuclear genomes probably evolve at a different rate and in very different ways.

### Mitochondrial Genome Size is Variable

Despite this high conservation of synteny, the comparison of the entire mt genomes highlights a wide genomic variability, in particular in non-coding sequences, which explains the difference in size. In the *Lachancea* genus, mt genome sizes vary considerably ranging from 23,584 bp to 51,525 bp in *L. thermotolerans* and *L. kluyveri*, respectively. This size variation is correlated with either the relative size of intergenic sequences or the intron content (Table 1). By contrast to the genomes of the *Nakaseomyces* clade, we did not observe a relation between the number of GC clusters and the size of these genomes (data not shown) [23]. Intergenic regions range from 23.1% in *L. mirantina* to 58% in *L. kluyveri*. The intron content of *COB*, *COX1* and *LSU* genes is very variable. The *COB* gene harbors introns in *L. kluyveri*, *L. mirantina* and *L. meyersii* (Fig. S1). The first intron is present in the three species whereas the second is only present in *L. kluyveri* and *L. meyersii*. The difference in the intron content is even more pronounced in the case of the *COX1* gene. The number of introns varies from 2 to 6 in *L. nothofagi* and *L. meyersii*, respectively (Fig. S1). With the exception of the first intron found in the *COB* gene, all introns encode endonucleases belonging to the LAGLIDADG family of group I introns [27]. Introns, which do not encode any endonuclease, were also detected in *LSU* gene of all species but *L. thermotolerans* and *L. nothofagi* (Fig. 1 and Fig. S1).

All in all, these data show that mitochondrial genome evolution of species of the *Lachancea* genus is mainly related to the intron mobility and intergenic region variation. This is a common rule of the plasticity of mt genomes within and among yeast species. The exploration of mt genomes of the same species (*L. kluyveri*) led to the same conclusion [2]. In addition, similar observations were reported in closely related species [20] as well as in more distant related species [23].

### Phylogenetic Relationship

To get a better insight into the evolution of the *Lachancea* genus, we investigated the phylogenetic relationship among the species based on our mitochondrial data. The phylogenetic analysis was based on the concatenation of coding gene sequences representing 6711 positions. A Neighbor-Joining tree was constructed based on these segregating sites and *Kluyveromyces lactis* (CBS 2359) was used as an outgroup (Fig. 2). This analysis confirmed that the species are closely related. The mitochondrial tree topology showed that the *Lachancea* genus is divided into three subgroups with a bootstrap support value of almost 100%. The first subgroup contains five species (*L. meyersii*, *L. fantastica*, *L. nothofagi*, *L. dasiensis* and *L. thermotolerans*). The second and third subgroups contain *L. mirantina* and *L. kluyveri*, respectively. *L. kluyveri* is basal to the genus with a high boostrap value. This species is the most divergent of the genus.

Previous phylogenetic analysis based on nuclear rDNA sequences (the D1/D2 domains of the 26S rRNA and 5.8S ITS region) already allowed the exploration of the relationship between *Lachancea* yeasts [4], [6], [7], [8], [9]. Overall, there is a high congruence of the topology found in these studies and the one we previously described. For example, *L. kluyveri* has been shown to be the most divergent species and to occupy a special position on the phylogenetic tree [4]. However, there is a minor conflict regarding the positioning of some species, which are in the first subgroup containing *L. meyersii*, *L. fantastica*, *L. nothofagi*, *L. dasiensis* and *L. thermotolerans*.

### Pattern of Gene Evolution and Selection

To explore the pattern of gene evolution, DNA sequence diversity in the coding region was compared between the seven *Lachancea* species. A total number of 1243 polymorphic positions showed a nucleotide substitution. The frequency of polymorphism is 0.0696 per bp on average. Among the 1119 SNPs identified in coding genes, 649 are non-synonymous and 470 are synonymous.

Selective constraints present in the mt genomes were then quantified by estimating the ratio of non-synonymous (dN) to synonymous (dS) substitution rates (ω  =  dN/dS). We calculated the average ω ratio in each of the coding genes (Fig. 3). Interestingly, dN was lower than dS, signature of strong purifying selection of mitochondrial genes (Fig. S2). In all the genes, median values of ω are lower than 1 (Fig. 3). Nevertheless the values differed from one gene to another. The *ATP6*, *COX3* and *VAR1* genes were found to be characterized by a dN/dS ratio, which is well above average, giving a median value of 0.15, 0.13 and 0.63 respectively. This finding might potentially be attributable either to positive selection or to a reduced constraint level on these genes (i.e. relaxation of the purifying selection). The results observed for *VAR1* gene is interesting because we observed an exceptionally low dS value (the lowest in this set) and high dN value than average (the highest in this set). The same results were obtained within the *L. kluyveri* species. In fact, the *VAR1* gene encodes a mitochondrial ribosomal protein and shows a scattered pattern of distribution among hemiascomycetous yeasts [21]. Indeed, *VAR1* is not found in the mitochondrial genome of species such as the closely related yeasts *C. parapsilosis, C. orthopsilosis* and *C. metapsilosis* [26]. Therefore, the relaxation of the purifying selection is probably related to a reduced functional constraint.

### Conclusion

These comparative data illuminate the influence of evolutionary forces that shape mitochondrial genome variation within and between species of the *Lachancea* genus. In fact, similar conclusions can be drawn about mt genome evolution among yeasts species of the *Lachancea* genus and within a single species: *L. kluyveri*. Genomes are completely syntenic across the whole genus even if genome sizes are very variable. The high plasticity of the genomes is related to intron content as well as intergenic region variation. The intron content varies in intron type, intron size and the presence or not of intron-coded endonucleases. The size of intergenic regions varies considerably and mutation rate in these regions is very high. This fact stands in contrast to the highly conserved coding-region. The pattern of variation observed among the mitochondrial genes is consistent with purifying selection across the whole genus, with the noteworthy exception of the *VAR1* gene.

## Supporting Information

Figure S1 Intron variability in *COX1* (A), *COB* (B) and *LSU* genes (C). All the coding introns found in these *COX1* and *COB* genes belong to the LAGLIDADG superfamily of group I introns and are presented in the form of orange circles. Numbers of circles depend on the number of LAGLIDADG motifs. Numbers are the coordinates of the corresponding protein sequence for *COX1* and *COB* and nucleotide sequence for *LSU*.
(EPS)

Figure S2 Box-plot comparisons of dN and dS substitution rates estimated in the various mt genes, based on pairwise alignments.
(EPS)
